# Patient-Reported Barriers to Ongoing Engagement in Office-Based Buprenorphine Treatment in a Semi-rural Missouri Clinic: A Cross-Sectional Survey

**DOI:** 10.7759/cureus.117867

**Published:** 2026-10-13

**Authors:** Jay Patel, Nathan Ngo, Kaley Hildebrand, Filomena J Galdikas, Nauman Ashraf

**Affiliations:** 1 College of Osteopathic Medicine, Kansas City University, Kansas City, USA; 2 Psychiatry, Freeman Health System, Joplin, USA; 3 Psychiatry, Kansas City University, Kansas, USA

**Keywords:** addiction psychiatry, buprenorphine, missouri, opioid use disorder (oud), social determinants of health (sdoh)

## Abstract

Access to evidence-based treatment for opioid use disorder (OUD) remains limited, and barriers to sustained engagement in office-based opioid treatment (OBOT) may vary across communities. This study aimed to characterize patient-reported barriers to accessing and remaining in OBOT in a semi-rural Missouri community. A single-center, cross-sectional descriptive survey at the Ozark Center clinic in Joplin, Missouri, was conducted among patients receiving OBOT for OUD. Approximately 170 patients were enrolled in the OBOT program during the study period, although not all patients were offered the survey due to scheduling and clinic workflow variability. The anonymous, paper-based 11-item survey was completed by 49 patients, with participation optional for each item and therefore variable responses across questions. Among respondents, 44/49 (89.8%) reported needing treatment, 44/49 (89.8%) reported feeling comfortable discussing substance use with healthcare professionals, and 47/49 (95.9%) reported encouragement from family, friends, or community members. Eight of 49 (16.3%) reported having difficulty finding a treatment clinic. Reported barriers to accessing treatment included cost (n=14), insurance issues (n=13), and transportation availability (n=13). The most frequently reported barriers to maintaining treatment were work or other obligations (n=30), clinic hours (n=20), feeling judged or looked down on (n=18), clinic location (n=16), and travel distance (n=15). Additionally, 21 participants reported experiencing stigma related to medications for OUD. These exploratory findings are limited by the small, nonuniform survey distribution, self-reported data, and inclusion of patients already engaged in treatment, and therefore may not reflect barriers experienced by individuals who have not initiated or discontinued OBOT. Larger, multisite studies including patients outside active treatment are needed to further characterize barriers to OUD treatment initiation and retention.

## Introduction

Although there are many resources available for addiction disorder treatment, many patients do not seek treatment. If a patient seeks treatment but relapses during the course, they may consider it a personal failure and not make future attempts at seeking help. This makes it extremely challenging for patients to improve their health status. When an overview of 12 systematic reviews was conducted, it grouped the barriers and facilitators to accessing treatment into three distinct levels: individual, social, and structural [1]. The majority of barriers and facilitators were structural. These included things like inadequate access, lack of qualified workers, policies that favor enforcement rather than harm reduction, and certain legal barriers. Similar results were found when a systematic review analyzed 40 studies on the barriers to using buprenorphine/naloxone and naltrexone for opioid use disorder (OUD) [2]. The study identified key obstacles at the patient and provider levels, including stigma, cost, limited provider training, and restrictive policies. Patients reported misconceptions about treatment and financial barriers, while providers cited concerns about medication misuse and time constraints. 

Marginalized populations that struggle with addiction often have an added disadvantage that makes this issue even more complex. A study in San Francisco [3] showed a strong, bidirectional correlation between feelings of guilt/shame and methamphetamine use among 110 sexual minority cisgender men. Researchers in this study made sure that the results were independent of depression symptoms to eliminate clinical depression as a confounding variable. 

Although extensive research has been done to identify barriers to accessing treatment, results vary based on the location. For instance, a study was done to identify barriers in continuing treatment after being discharged from a detox center in Boston [4]. At this location, patients expressed issues with a lack of continuity of care, limited number of detox centers, unstable housing, and a lack of treatment options. In contrast, a similar study based in West Virginia [5] identified the barriers to treatment for those injecting drugs. In West Virginia, key issues included low threshold for dismissal, lack of comprehensive support, and inadequate management for withdrawal symptoms. Demographic and political/economic factors within a set area can also influence which barriers patients experience. 

The present study aims to characterize patient-reported barriers to ongoing engagement in office-based opioid treatment (OBOT) among patients receiving care at the Ozark Center for Addiction Medicine in Joplin, Missouri. 

## Materials and methods

The project was approved by the Institutional Review Board at the Ozark Center in Joplin, Missouri. Approximately 170 patients were enrolled in the office-based opioid treatment (OBOT) program at the time of data collection. Due to scheduling variability and workflow factors, not all patients were offered a survey. 

This single-center, cross-sectional descriptive survey included patients diagnosed with opioid use disorder coming into the addiction clinic who were receiving buprenorphine treatment through the addiction clinic at Freeman Health System. Patients were recruited through routine clinic encounters during the data collection period (August 2025-October 2025), resulting in non-probability convenience sampling.

No formal a priori sample size calculation was performed because this was an exploratory descriptive study based on the patients available during the data-collection period and the feasibility of survey administration. 

A total of 49 patients completed the anonymous questionnaire. Specific information related to patient demographics for those who completed the survey was not collected to protect anonymity. The questionnaire was developed by members of the study and reviewed by Dr. Nauman Ashraf. The survey was not a validated instrument, and findings should be interpreted as exploratory. No formal pilot testing or psychometric validation was performed. The survey consisted of 11 questions, with survey items assessing stigma (Q10 - experiencing stigma), logistical factors (Q6 - able to find info), transportation issues, financial limitations (Q8 - barriers to accessing treatment), clinic accessibility (Q3 - trouble finding clinic), and comfort discussing treatment with healthcare professionals (Q4 - comfortability with providers). Most of the question responses were defined by either a "yes" or "no" answer choice, two were structured as a "choose all that apply", and another one was given a free-response approach. The survey was distributed on paper, and patients marked their answers in pen. Survey responses were electronically transcribed into a compiled dataset for analysis. Strict confidentiality protocols were followed as per IRB protocol. 

Response frequencies were tabulated and organized into conceptual categories. Analyses were descriptive only. Categorical responses were summarized using frequencies and percentages, and no inferential statistical tests or multivariable analyses were performed. Because participation in each item was optional, response totals vary slightly across questions. Item-level missing responses were not imputed. Distributors of the survey were instructed to clarify with age-appropriate terminology or alternative slang if the patient did not understand certain language within the survey. 

## Results

Most participants, 44 out of 49, indicated that they needed treatment (Q2 - need treatment); 44 felt comfortable discussing their substance use with healthcare professionals (Q4 - comfortable with providers), reflecting generally positive prior perceptions of provider experiences. Forty-seven reported support from friends, family, or community members (Q5 - encouraged by family/community), suggesting that social support networks were present for the majority of individuals. Eight reported difficulties finding a clinic (Q3 - trouble finding clinic) (Figure 1).

**Figure 1:**
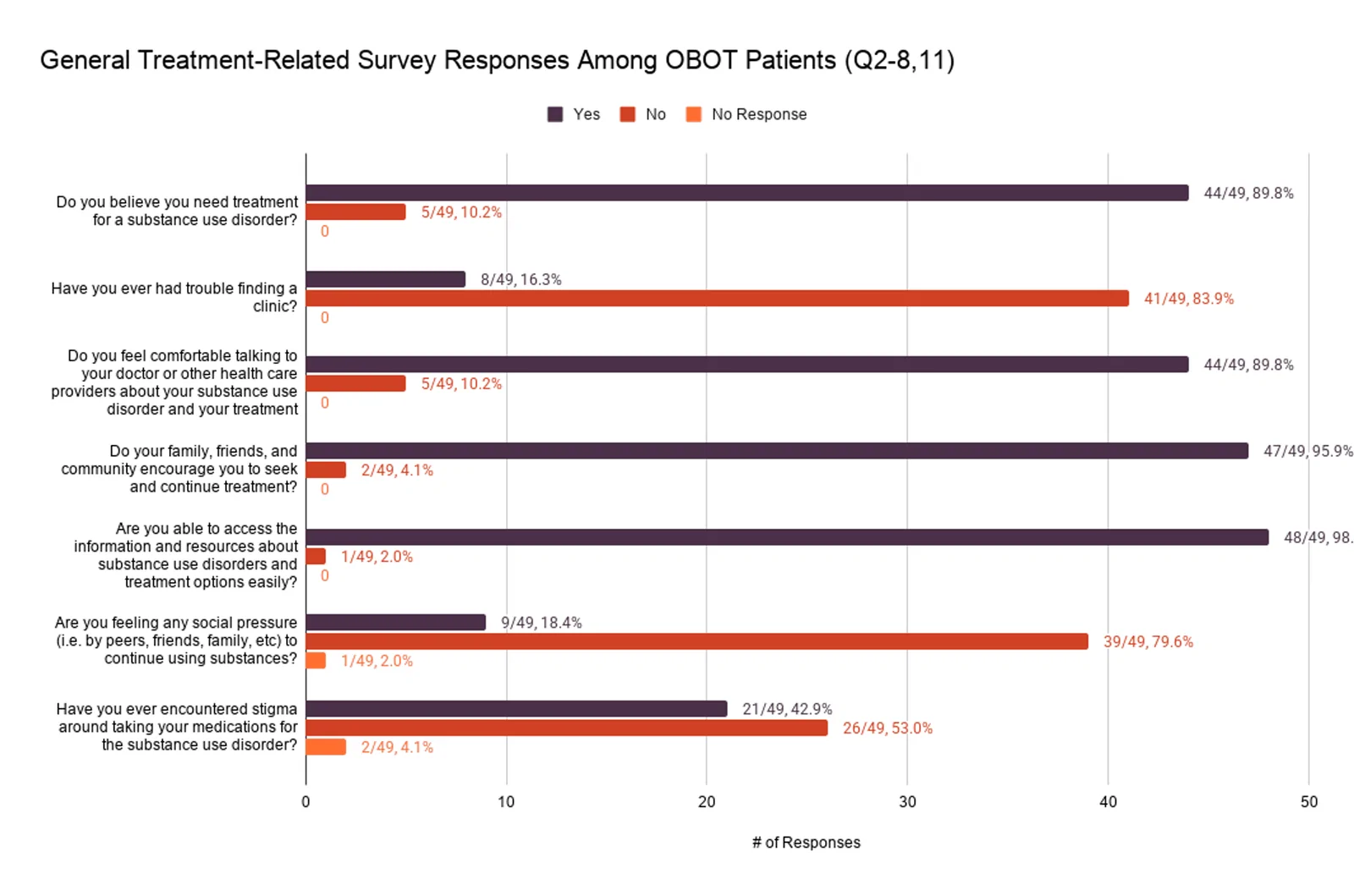
General treatment-related survey responses among OBOT patients OBOT - office-based opioid treatment

Barriers to accessing medical attention (Q8 - barriers to accessing treatment) included cost (n=14), insurance issues (n=13), and transportation availability (n=13). These responses indicate that financial and transportation-related barriers were reported by some participants. Reported barriers to ongoing treatment engagement (Q9-Barriers to Staying on Treatment) were most frequently related to work or other obligations (n=30), followed by clinic hours (n=20), feeling judged or looked down on (n=18), clinic location (n=16), and travel distance (n=15) (Figure 2). 

**Figure 2:**
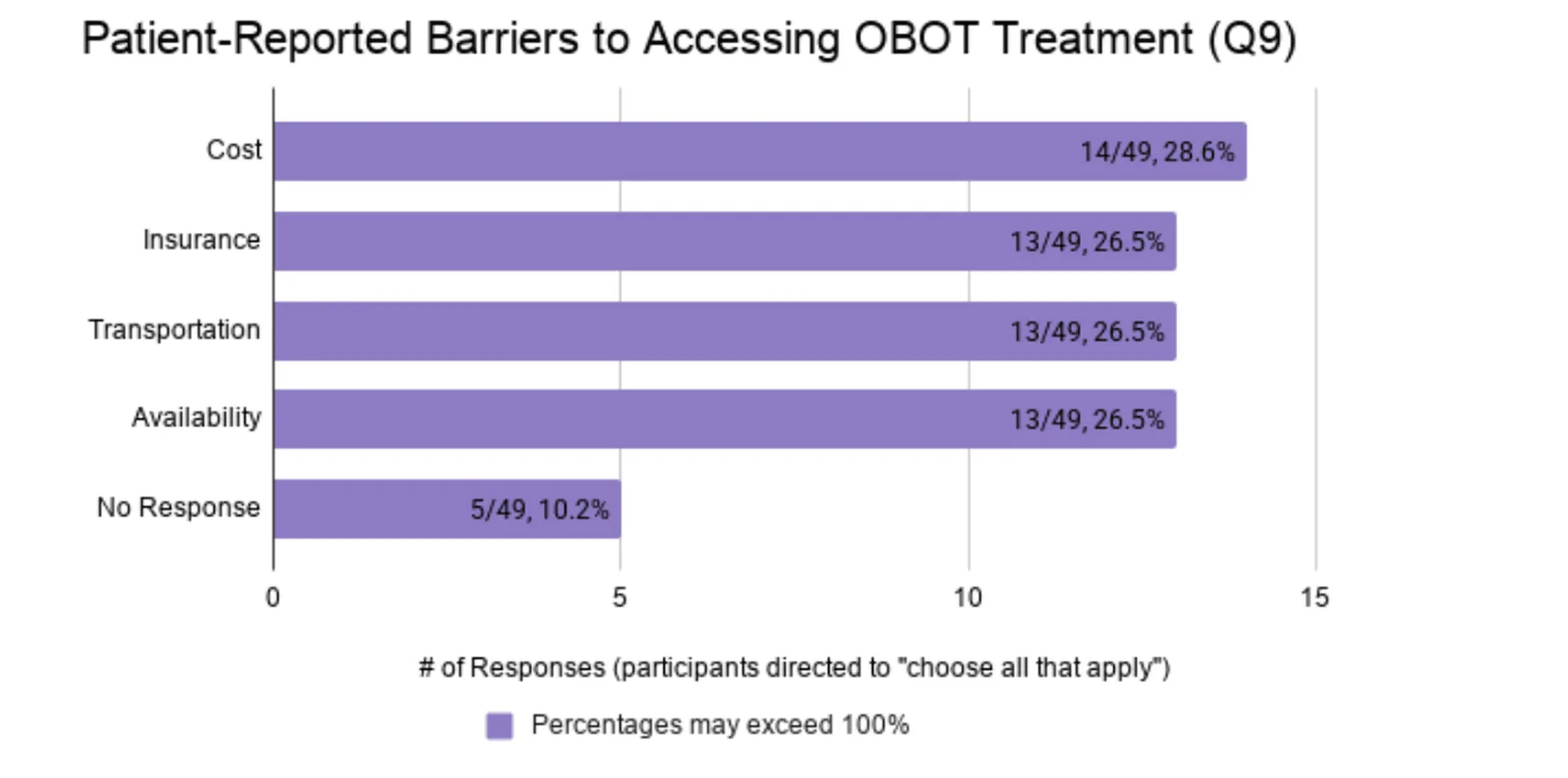
Patient-reported barriers to accessing OBOT treatment OBOT - office-based opioid treatment

Less common challenges (Q9 - barriers to staying on treatment) included lack of peer support (n=8), side effects (n=8), and medication being stolen or misused (n=6). In addition, 21 participants reported experiencing stigma related to taking medications for opioid use disorder (Figure 3). 

**Figure 3:**
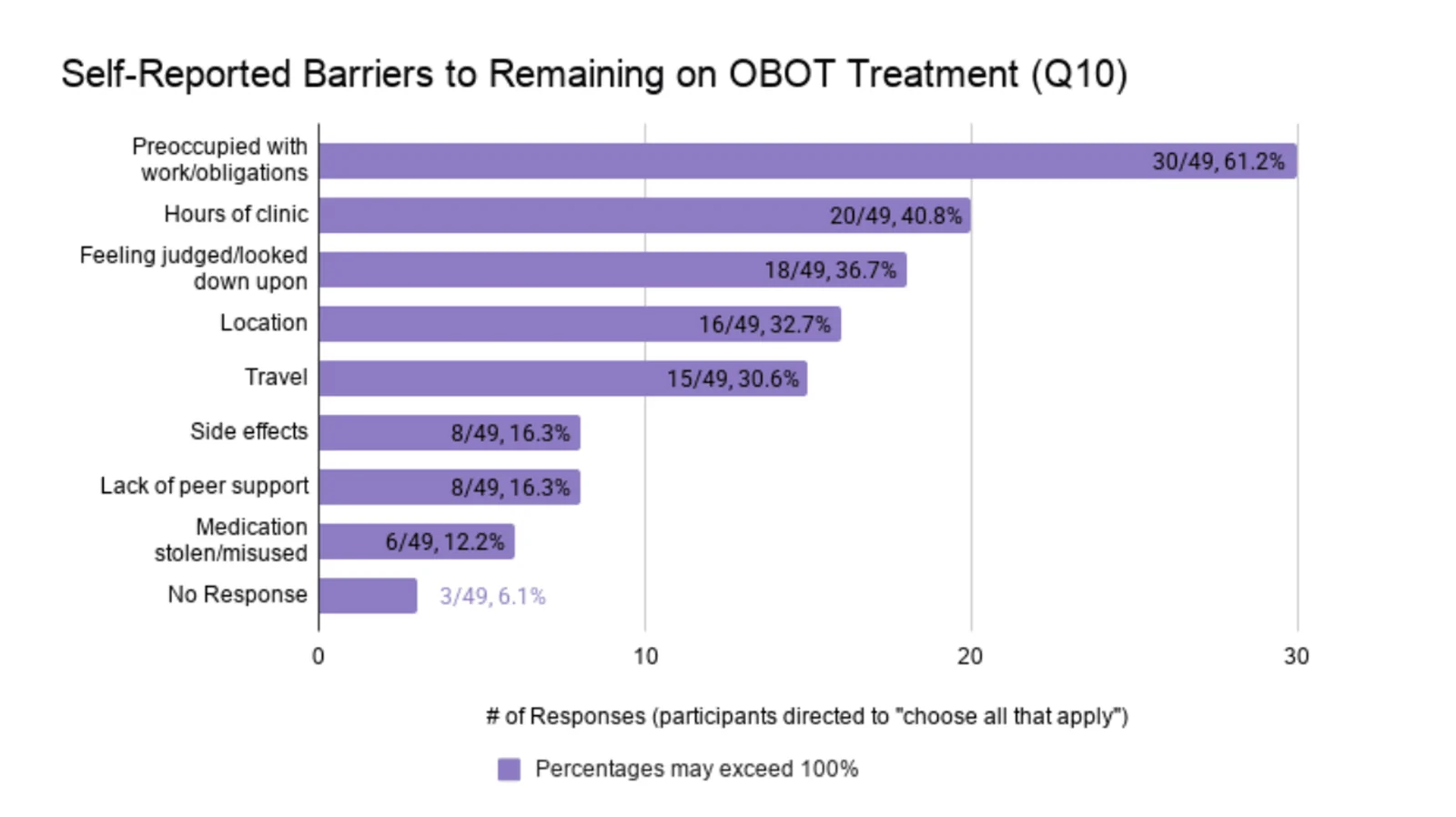
Self-reported barriers to remaining on OBOT treatment OBOT - office-based opioid treatment

## Discussion

The most prominent barriers to OBOT engagement among surveyed patients in this community were work or other obligations, clinic hours, feeling judged, and transportation-related challenges, including clinic location and travel distance. Financial constraints such as cost and insurance difficulties were also meaningful barriers. This is further expounded upon through the geographical context behind Joplin, a semi-rural town in Missouri. As recognized by others, rural conditions can accentuate already difficult conditions and worsen access to proper treatment [6].

Although 47 of 49 participants reported encouragement from family, friends, or community members, a substantial proportion (21 of 49) simultaneously reported experiencing stigma toward taking medication for opioid use disorder (MOUD). These findings suggest that support for treatment and stigma toward MOUD can coexist, arising from interpersonal, societal attitudes, or self- stigma. Encouragement to seek treatment does not necessarily eliminate internalized or societal stigma associated with opioid use disorder or MOUD. A nationally representative survey has also examined associations between opioid treatment availability and opioid related stigma [7]. And given the semi-rural nature of Joplin, there is a required consideration for a potential low spatial availability for this specific patient population and their unique needs. 

Among patients already engaged in OBOT, the reported barriers were more frequently related to maintaining ongoing treatment rather than to locating a treatment clinic. The high proportion of respondents reporting comfort with healthcare professionals and the relatively low frequency of reported difficulty locating a treatment clinic suggest that, within this enrolled sample, commonly reported barriers were often more related to ongoing participation than to locating care. 

However, several limitations should be recognized. For instance, because these were self-reported surveys taken at a given time, it is difficult to establish causal relationships between the identified barriers and treatment retention. Longitudinal studies could better characterize how reported barriers influence engagement over time. Limitations of this study include the smaller sample size. Although we received insight from 49 patients, the small sample size limits precision and reduces the ability to assess variation across patient characteristics or clinical subgroups. In addition, this study did not collect demographic information for the patients. Including patient demographics like age, gender, sexuality, and marital status would provide a more accurate understanding of those who live in the community and how their circumstances correlate with the barriers they face. Collecting additional information, including other comorbid conditions, duration of treatment, and any relapses these patients had gone through, would better help understand the patient population. Allowing patients to also submit a response with their yes/no answer to the survey questions would also provide greater insight. And because participants were already receiving buprenorphine treatment, the study may be subject to selection bias and cannot characterize barriers experienced by individuals who never initiated treatment or discontinued treatment before the survey was administered. Also, the nonuniform distribution of the survey may have introduced the selection bias mentioned earlier because patients who were offered and completed the survey may have differed systematically from those who were not offered or did not complete it. Moreover, the questionnaire was developed by the study team and was not a validated instrument, which could potentially limit measurement reliability and comparability with other studies. 

## Conclusions

Although non-uniform survey distribution related to clinic workflow variability and the small number of completed surveys may limit generalizability, this study provides preliminary insight into patient- reported barriers to OBOT in a semi- rural Missouri community. These findings can inform the design of future larger, multi- site studies to better define regional barriers to opioid use disorder treatment and to develop strategies that optimize long- term engagement in care. The findings of the study can also provide insight into quality improvement initiatives to address substance use disorder in Joplin, MO, and other semi- rural communities like it in the United States.

Overall, addressing the ongoing issue of substance use disorder in the United States through programs such as OBOT is vital in reducing the number of deaths per year from overdose. These exploratory findings suggest that work schedules, clinic hours, transportation, cost, insurance barriers, and stigma may affect ongoing engagement in OBOT among patients already enrolled in care. These findings may help inform quality improvement efforts among patients receiving OBOT, while larger multisite studies are needed to determine whether these patient findings extend to other populations. 

## Figures and Tables

**Figure 4:**
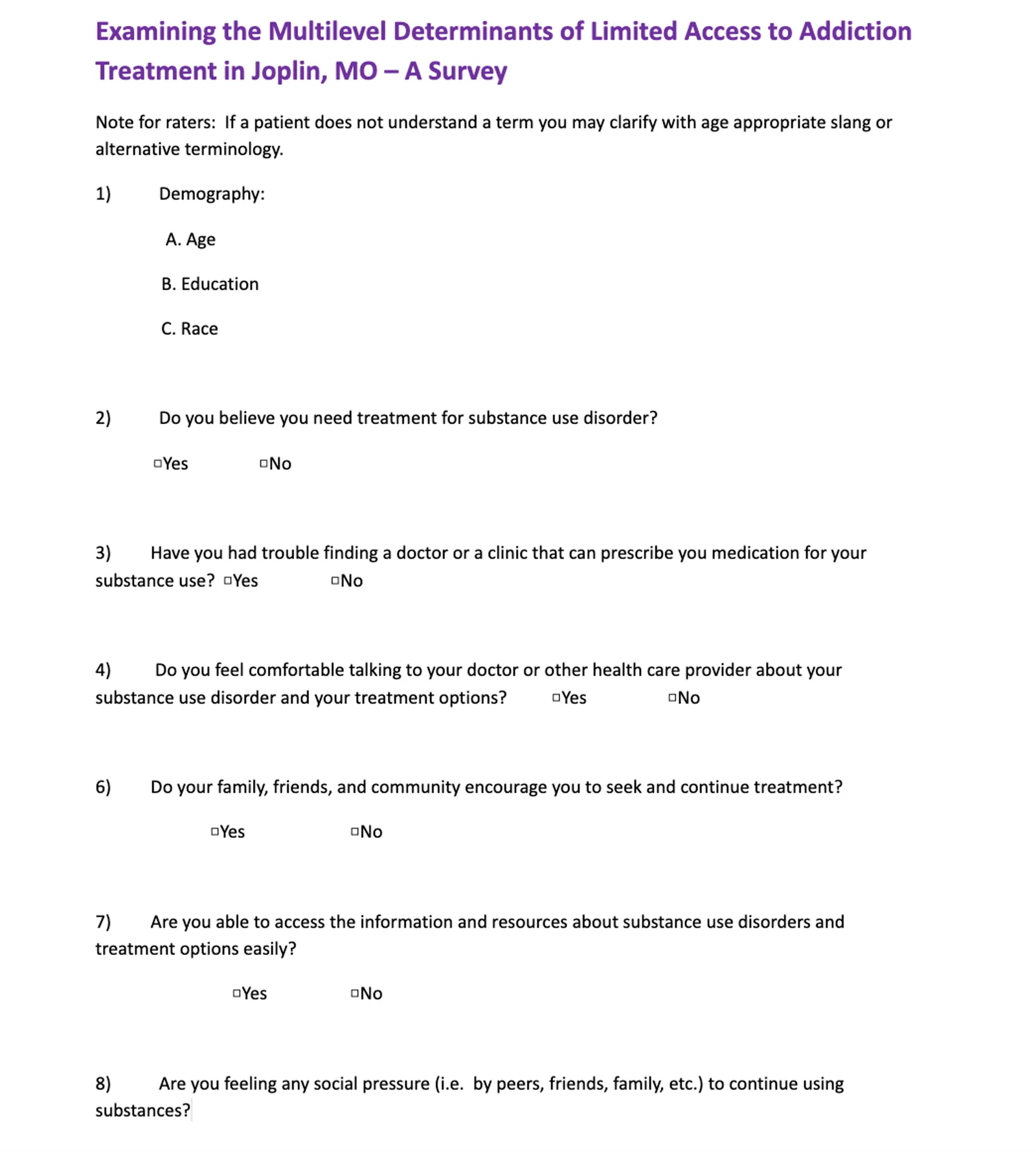
Page 1 of 2 of the survey given to patients Q1 is free response, while Q2-8 are yes/no and are represented in Figure 1. Q8's yes/no boxes overflow onto page 2 of 2.

**Figure 5:**
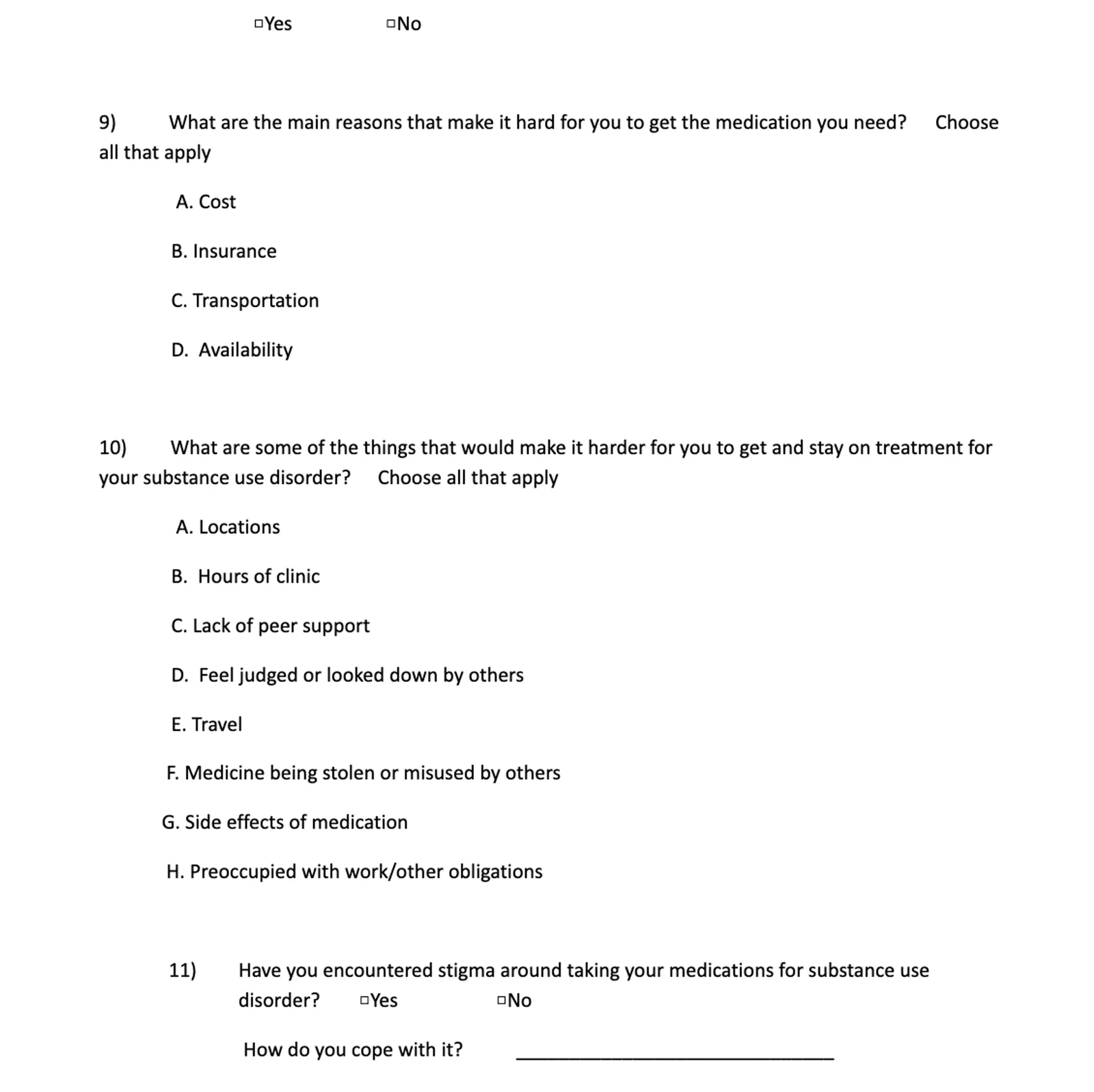
Page 2 of 2 of the survey administered to patients The yes/no boxes on the top of the page correspond to Q8 on page 1 of 2. Q9 and Q10 are "choose all that apply", so patients circled all statements that applied to them with results corresponding to Figure 2 and Figure 3, respectively. Q11 is a yes/no question with results shown in Figure 1, with an optional free response to provide a description on how patients cope with stigma.
